# Identifying key multi-modal predictors of incipient dementia in Parkinson’s disease: a machine learning analysis and Tree SHAP interpretation

**DOI:** 10.3389/fnagi.2023.1124232

**Published:** 2023-06-30

**Authors:** G. Peggy McFall, Linzy Bohn, Myrlene Gee, Shannon M. Drouin, Harrison Fah, Wei Han, Liang Li, Richard Camicioli, Roger A. Dixon

**Affiliations:** ^1^Department of Psychology, University of Alberta, Edmonton, AB, Canada; ^2^Neuroscience and Mental Health Institute, University of Alberta, Edmonton, AB, Canada; ^3^Department of Medicine (Neurology), University of Alberta, Edmonton, AB, Canada; ^4^Department of Computing Science, University of Alberta, Edmonton, AB, Canada; ^5^Department of Chemistry, University of Alberta, Edmonton, AB, Canada

**Keywords:** Parkinson’s disease, dementia, risk factors, biomarkers, random forest classifier, Tree SHapley Additive exPlanation

## Abstract

**Background:**

Persons with Parkinson’s disease (PD) differentially progress to cognitive impairment and dementia. With a 3-year longitudinal sample of initially non-demented PD patients measured on multiple dementia risk factors, we demonstrate that machine learning classifier algorithms can be combined with explainable artificial intelligence methods to identify and interpret leading predictors that discriminate those who later converted to dementia from those who did not.

**Method:**

Participants were 48 well-characterized PD patients (*M*_baseline age_ = 71.6; *SD* = 4.8; 44% female). We tested 38 multi-modal predictors from 10 domains (e.g., motor, cognitive) in a computationally competitive context to identify those that best discriminated two unobserved baseline groups, PD No Dementia (PDND), and PD Incipient Dementia (PDID). We used Random Forest (RF) classifier models for the discrimination goal and Tree SHapley Additive exPlanation (Tree SHAP) values for deep interpretation.

**Results:**

An excellent RF model discriminated baseline PDID from PDND (*AUC* = 0.84; normalized *Matthews Correlation Coefficient* = 0.76). Tree SHAP showed that ten leading predictors of PDID accounted for 62.5% of the model, as well as their relative importance, direction, and magnitude (risk threshold). These predictors represented the motor (e.g., poorer gait), cognitive (e.g., slower Trail A), molecular (up-regulated metabolite panel), demographic (age), imaging (ventricular volume), and lifestyle (activities of daily living) domains.

**Conclusion:**

Our data-driven protocol integrated RF classifier models and Tree SHAP applications to selectively identify and interpret early dementia risk factors in a well-characterized sample of initially non-demented persons with PD. Results indicate that leading dementia predictors derive from multiple complementary risk domains.

## 1. Introduction

Parkinson’s disease (PD) is a complex, multisystem disorder characterized by pathological degeneration of nigrostriatal dopaminergic neurons and the presence of Lewy bodies (Gomperts, 2016; Aarsland et al., 2017). Although PD is primarily characterized as a movement disorder, it includes a wide variety of non-motor symptoms (Marinus et al., 2018), including accelerated cognitive decline leading to impairment or dementia (Guo et al., 2021). PD patients are two to six times more likely to develop dementia than healthy controls who are asymptomatic for neurodegenerative disease. Notably, although not all PD patients develop dementia, researchers estimate long-term conversion rates at between 50 and 80% of newly diagnosed PD patients (Marinus et al., 2018; Guo et al., 2021). Parkinson’s disease dementia (PDD) includes not only motor and cognitive impairment, but is also associated with an increased risk of additional adverse outcomes. These include reduced physical function, poorer quality of life, increased caregiver burden, and increased health-related costs (Svenningsson et al., 2012). Early identification of individuals living with PD who are at an increased risk for dementia may enable early and targeted interventions that offset or delay cognitive decline or impairment and mitigate some of the additional negative outcomes of PDD.

Recent studies have investigated multiple independent (or candidate) predictors of dementia in diagnosed PD patients. To date, these predictors represent several risk factor and biomarker domains (Vasconcellos and Pereira, 2015; Delgado-Alvarado et al., 2016; Gomperts, 2016; Hanagasi et al., 2017). Examples that increase risk of conversion to dementia in PD patients include (a) older age (Vasconcellos and Pereira, 2015; Cereda et al., 2016), (b) lower performance in cognitive measures such executive function, episodic memory, and speed/inconsistency (de Frias et al., 2012; Hanagasi et al., 2017), (c) metabolomics-based and lipidomics-based biomarker panels (Han et al., 2017; Buzatto et al., 2021), (d) orthostatic hypotension (defined as the drop of 10 mmHg blood pressure when standing as compared to supine; Anang et al., 2014), and (e) ventricular dilation (Camicioli et al., 2011). A recent systematic review also identified several clusters of baseline factors associated with PDD diagnosis approximately 4.4 years later, including age (older), common clinical PD factors (gait disturbances, motor function disorder), rapid eye movement (REM) sleep behavior disorders, orthostatic hypotension, and hallucinations (Guo et al., 2021). Interestingly, this review identified several additional factors that were correlated with higher risk of PD-related cognitive impairment (not yet dementia), including age of onset, genetic risk (*Apolipoprotein E* [*APOE*], *microtubule associated protein tau* [*MAPT*]), Unified Parkinson’s Disease Rating Scale (UPDRS) III scores, and anxiety. In cross-sectional research, risk factors associated with PDD include: (a) male sex (Cereda et al., 2016), (b) higher homocysteine level (Song et al., 2013), and (c) higher white matter hyperintensities load (Dadar et al., 2020).

Given that a diverse set of risk predictors have been associated with the gradual emergence of PDD, a broad prediction analysis that includes factors from multiple modalities evaluated simultaneously and competitively may identify markers that contribute most to the early detection of PDD vulnerability. An appropriate prospective design would include a baseline sample of PD participants with no dementia (PDND) from which two subgroups emerge at later time points—those confirmed as having remained as PDND and those diagnosed as having developed PDD in the interim. At baseline, the latter unobserved subgroup could be designated as PD incipient dementia (PDID). Relevant baseline measures would sample from the above list of associated factors, as well as new candidates from domains of known PD dementia risk. Pertinent analytics would feature the capacity to evaluate multiple predictors simultaneously in a computationally competitive context, determine the most important predictors, and interpret the direction and magnitude of the effects. In addition, the analytics would be effective in both larger databases and smaller clinical samples. In the present study, we integrate these methodological and analytic characteristics to demonstrate their application to a clinical sample of initially non-demented PD patients who are followed over a 3-year period and are then diagnosed as either PDD or PDND.

Several recent studies have tested multiple risk factors and biomarkers to distinguish PDND from PDID (Liu et al., 2017; Dawson et al., 2018; Phongpreecha et al., 2020). Overall, these studies featured relatively large samples but tested relatively few dementia predictors (ranging from 6 to 16). Although the predictors represented a promising but selected range of potential PD-related dementia risk factors, there was some overlap (e.g., age, sex, education) across studies. For example, the Montreal Parkinson Risk of Dementia Scale (MoPaRDS) consisting of eight risk factors [sex, age, mild cognitive impairment (MCI), bilateral disease onset, REM sleep behavior disorder, hallucinations, falls and/or freezing, orthostatic hypotension] distinguished PDND from PDID, with the PDD outcome at a 4.4-year interval (Area Under the receiver operating characteristic Curve [*AUC*] = 0.88 (Dawson et al., 2018), see also (Bohn et al., 2023)). In another example, Liu et al. (2017) followed a large sample of PD patients for approximately 9 years, testing seven baseline PD dementia risk factors (age of onset, mini-mental state exam, education, motor exam score, sex, depression, β-glucocerbrosidase [GBA]) for those that discriminated participants who developed dementia (PDID at baseline) from those who did not (PDND; *AUC* = 0.88). In a broader investigation, Phongpreecha et al. (2020) used a classification model including baseline factors—10 biological (age, education, sex, disease duration, depression, levodopa equivalent daily dose, symptom progression, *APOE, GBA, MAPT*) and 6 cognitive (Montreal Cognitive Assessment [MoCA], total recall, delayed recall, recognition discriminability, Trail A and B)—to discriminate PDND from PDD up to 4 years later. Notably, they observed that the cognitive variables (*AUC* = 0.90) were stronger predictors than their sampling of biological factors (*AUC* = 0.77). Finally, Schrag et al. (2017) grouped 22 baseline dementia-related risk factors by domain and tested the association of each domain with PDD 2 years later. Tested models included risk domains of (a) clinical, (b) imaging, (c) cerebrospinal fluid, and (d) independent variables from all three. Notably, no model simultaneously tested all 22 risk factors. In univariate analyses, the five variables most strongly associated with cognitive decline were age, smell, REM sleep behavior disorder, CSF amyloid β, and caudate uptake (*AUC* = 0.80). Overall, findings from these studies suggest that multiple risk factors from several domains (e.g., demographic, neurocognitive, gait, metabolic, imaging) may distinguish PDND from PDID subgroups in non-demented PD patients.

Accumulating results indicate the potential for heterogeneous risk factors to be associated with the differential emergence of dementia in PD patients. In this study, we integrate a machine learning classifier approach [random forest (RF) algorithm for leading predictor identification] and an explainable artificial intelligence method [Tree Shapley Additive exPlanation (Tree SHAP) for informed interpretation] to simultaneously test a large number and diverse range of predictors representing multiple established domains of dementia risk in PD. Similar multi-variable biomarker prediction approaches to longitudinal data have been suggested for related complex and dynamic neurodegenerative diseases (Fotuhi et al., 2009; Aarsland and Kurz, 2010; Sapkota et al., 2018; Badhwar et al., 2020; Wang et al., 2022). Machine learning approaches use computer systems that apply algorithms and quantitative models to analyze and draw inferences from patterns in big or high dimensional data. The competitive computational context of machine learning prediction models promotes the identification of the most important predictors from a large number of risk factors tested in relation to all other risk factors (i.e., considering factor dependences and interactions). An integrative analytic workflow via Tree SHAP provides interpretation of identified predictors (e.g., relative magnitude, direction).

The main research goal of the current study was to apply machine learning technology, specifically RF classifier, to an extensive multi-factorial battery of baseline dementia risk factors. We selected RF classifier as it includes two important capacities: (a) it is designed to test competitively a large number of multi-domain predictors and (b) it has the capacity to do so for samples with large or small numbers of participants. To confirm that RF classifier was the best model for these data, we ran comparisons using Logistic Regression and Gradient Boosting (GB) models. These prediction analyses were followed by Tree SHAP applications for deeper interpretation. The purpose was to identify the best predictors that distinguish among a baseline cohort of diagnosed PD participants, all of whom were initially non-demented, but some of whom converted 3 years later to PDD and some of whom did not. We refer to these two subgroups as PDID and PDND, respectively.

## 2. Materials and methods

### 2.1. Participants

Participants were PD patients with no dementia (*n* = 52) recruited between 2003 and 2009 from the University of Alberta Movement Disorders Clinic, the Parkinson’s Society of Alberta, and community neurologists. Prior to enrollment in the study, PD patients were evaluated by experienced neurologists to confirm PD diagnosis based on the presence of two of three typical signs of PD (rest tremor, bradykinesia, rigidity), consistent with UK Brain Bank Criteria (Gibb and Lees, 1988; Clarke et al., 2016). Potential recruits were excluded by the study neurologist (RC) if they met criteria for atypical Parkinsonism, had a clinical history of stroke, or an unstable health condition. Data for this single-site longitudinal study were collected for each participant at baseline, 18 months, and 36 months by the study neurologist and trained research staff. The private dataset is available upon reasonable request to RC. For the current study, participants were excluded if they were not available (i.e., dropped out or died) for the dementia classification protocol at 36 months (*n* = 4). The final sample consisted of 48 PD patients (*M*_baseline age_ = 71.5, *SD* = 4.8; 43.8% female; see Table 1 for a complete reporting of descriptive statistics). At baseline, all participants were classified as non-demented based on an assessment by the study neurologist (Camicioli et al., 2009). Specifically, they met all inclusion criteria and did not have cognitive problems sufficient to affect activities of daily living. At 36 months, 14 PD patients were clinically diagnosed with dementia and retrospectively classified as PDID at baseline. The remaining participants were classified as PDND (*n* = 34). All participants provided written informed consent and all data collection procedures were in full compliance with human research ethics.

**TABLE 1:** Baseline characteristics and predictors for PDND and PDID subgroups.

Characteristic *M* (*SD*)	Total sample	PDND	PDID	% Missing at baseline
N	48	34	14	0
PD duration (years)	8.9 (4.5)	8.6 (4.4)	9.5 (5.1)	0
UPDRS part III	16.6 (8.1)	14.4 (7.0)	21.7 (8.5)	0
Modified Hoehn and Yahr	2.2 (0.7)	2.0 (0.5)	2.6 (0.7)	0
Age (years) Range	71.6 (4.77) 65 – 84	70.0 (3.53) 65 – 77	75.4 (5.33)*** 66 – 84	0
Sex *n* (% Female)	21 (43.8)	13 (38.2)	8 (57.1)	0
Education (years)	14.1 (2.96)	14.3 (3.20)	13.6 (2.31)	0
Gait speed (standardized time in sec)	0.411 (1.59)	−0.076 (0.635)	1.59 (2.44)***	0
Gait steps (standardized number of steps)	0.420 (1.55)	−0.082 (0.568)	1.64 (2.36)***	0
Balance (standardized time; sec)	8.82 (4.40)	10.2 (3.82)	5.57 (4.09)***	0
Finger dexterity (number of taps in 60 sec)	114.8 (25.3)	121.6 (24.6)	98.2 (19.2)**	0
Visual acuity (feet)^a^	34.9 (15.5)	32.6 (13.4)	40.4 (19.2)	0
Smell (number correct out of 12)	6.60 (2.92)	6.94 (2.74)	5.79 (3.26)	2.1
APOE *n* (ε2 + , ε3ε3, ε4 +)^b^	(6, 35, 7)	(5, 25, 4)	(1, 10, 3)	0
MTHFR *n* (CC, CT, TT)	(20, 23, 4)	(14, 17, 3)	(6, 6, 1)	2.1
Third ventricle volume (cm^3^)^c^	1.08 (0.311)	0.989 (0.249)	1.34 (0.324)***	2.1
Fourth ventricle volume (cm^3^)^c^	1.25 (0.270)	1.26 (0.291)	1.21 (0.210)	2.1
White matter hyperintensities (cm^3^)^c^	2.54 (2.55)	2.33 (2.47)	3.09 (2.77)	2.1
Cortical thickness (mm)	2.31 (0.083)	2.33 (0.073)	2.25 (0.085)**	2.1
Systolic orthostatic hypotension (mm Hg)	8.38 (15.0)	6.84 (15.2)	13.3 (14.0)	12.5
Diastolic orthostatic hypotension (mm Hg)	2.00 (10.7)	0.69 (9.44)	6.20 (13.6)	12.5
Pulse pressure (mm Hg)	47.5 (8.33)	47.1 (8.34)	48.6 (8.52)	0
Heart rate (beats/min)	69.1 (9.85)	68.8 (9.24)	69.9 (11.5)	0
Creatinine (mg/dL)	84.6 (15.2)	87.6 (14.4)	77.4 (15.3)*	0
Homocysteine (mcmol/L)	13.5 (3.73)	13.4 (4.04)	13.7 (2.96)	0
Vitamin B12 (pmol/g)	294.6 (110.7)	293.1 (102.0)	298.1 (133.9)	0
Triglyceride (mmol/L)	1.12 (0.459)	1.07 (0.430)	1.22 (0.525)	2.1
Cholesterol HDL ratio (mg/dL)	3.61 (0.825)	3.49 (0.726)	3.89 (0.995)	2.1
Up-regulated metabolite panel	1.32 (0.532)	1.17 (0.414)	1.80 (0.608)***	12.5
Down-regulated metabolite panel	0.853 (0.647)	0.932 (0.710)	0.600 (0.281)	12.5
Immediate word recall (total correct)	37.2 (10.7)	39.8 (10.5)	31.0 (8.87)**	0
Trail A (sec)	50.7 (18.3)	44.1 (12.1)	66.9 (21.1)***	0
Trail B (sec)	139.5 (78.8)	113.1 (63.3)	203.8 (77.5)***	0
Simple reaction time (ms)	383.4 (101.7)	370.1 (73.3)	415.7 (148.8)	0
Four choice reaction time (ms)	1052.6 (251.7)	962.3 (141.0)	1258.9 (325.5)***	4.2
Depression (total out of 15)	1.87 (2.38)	1.38 (1.60)	3.07 (3.45)*	0
NPI-sleep (frequency × severity)	1.50 (2.75)	1.82 (3.04)	0.71 (1.73)	0
NPI-anxiety (frequency × severity)	0.54 (1.25)	0.47 (1.19)	0.71 (1.44)	0
Activities of Daily Living (% independence)	88.6 (8.80)	91.5 (4.69)	81.8 (12.3)***	0
Body mass index (kg/m^2^)	27.4 (5.14)	26.6 (4.17)	29.1 (6.82)	0
SMMSE (total out of 30)	28.1 (1.70)	28.6 (1.44)	26.8 (1.63)***	0
MoPaRDS (total out of 8)	3.04 (1.54)	2.56 (1.42)	4.21 (1.19)***	0

^a^What this person sees at 20 feet, an average person sees at x feet, x reported. ^b^ε2ε4 (n = 1) grouped with ε4 + . ^c^normalized using the proportional approach (area of interest/intracranial volume). PDND, Parkinson’s disease with no dementia; PDID, Parkinson’s disease with incipient dementia; PD, Parkinson’s disease; UPDRS, Unified Parkinson’s Disease Rating Scale; NPI, Neuropsychiatric Inventory Questionnaire; SMMSE, Standardized Mini Mental State Exam; MoPaRDS, Montreal Parkinson Risk of Dementia Scale. Baseline variables for PDID significantly differed from PDND *p < 0.05, **p < 0.01, and ***p < 0.001.

### 2.2. Dementia diagnosis

Dementia classification at 36 months was assessed by the study neurologist using the DSM-IV criteria (Camicioli et al., 2011). Briefly, participants were diagnosed with PDD if there was impairment in two cognitive domains plus functional impairment. Assessments were based on (a) clinical data, (b) independent interviews with the PD patient and an informant, (c) the Clinical Dementia Rating Scale (Morris, 1993), (d) Standardized Mini-Mental Status Examination (SMMSE) (Molloy and Standish, 1997), (e) the Dementia Rating Scale (Brown et al., 1999), and (f) the Short Blessed Information-Memory-Concentration Test (Fillenbaum et al., 1987).

### 2.3. Risk factor and biomarker predictors of PD incipient dementia

A pool of 38 baseline biomarkers and risk factors were used in analyses for predicting PDID (vs. PDND) in the PD baseline sample. These predictors were informally aligned with 10 domains of dementia risk. The demographic domain (*n* = 3) included *age* (in years), *sex* (male, female), and *education* (in years). The gait and motor function domain (*n* = 4) included *gait speed* (standardized average time in seconds for two trials of simple gait and two trials of dual task gait [naming male and female names]), *gait steps* (standardized average number of steps for two trials of simple gait and two trials of dual task gait [naming male and female names]), *balance* (standardized average time in seconds of keeping balance with eyes open and eyes closed with legs in different positions [e.g., one leg in front of the other]), and *finger dexterity* (average of left and right hand finger taps per minute). The sensory domain (*n* = 2) included *visual acuity* (measured using a reduced Snellen eye chart held 14 inches away from the participant’s face using both eyes and recorded as the number of feet a normal vision person can see in relation to what the participant can see at 20 feet) and *smell* (measured using the Brief Smell Identification Test, number correct out of 12 possible odorants (Doty et al., 1996). The genetic domain (*n* = 2) included *APOE* (rs429358, rs7412) and *methylenetetrahydrofolate reductase* (*MTHRF* rs1801133). *APOE* was categorized into the following three groups representing increasing dementia risk: ε2 + (ε2ε2, ε2ε3), ε3ε3, ε4 + (ε3ε4, ε4ε4). Due to low frequency and similarity of genetic risk, one ε2ε4 case was coded as ε4 + (Goldberg et al., 2020; Guo et al., 2021). *MTHRF* was categorized into the following three groups representing increasing dementia risk: CC, CT, TT. Original genotyping was conducted for these two candidate genes only; therefore, KEGG pathway and Gene Ontology analysis were not undertaken. The neuroimaging domain (*n* = 4) included *third ventricle volume*, *fourth ventricle volume, white matter hyperintensities*, and *cortical thickness* (average thickness of all brain area). All imaging measures, except cortical thickness, were corrected for intracranial volume (Sundermann et al., 2018). The cardiovascular domain (*n* = 4) consisted of *systolic orthostatic hypotension* (supine blood pressure minus standing blood pressure), *diastolic orthostatic hypotension*, *pulse pressure*, and *heart rate*. The candidate biomarker domain (*n* = 5) consisted of *homocysteine*, *vitamin B12*, *creatinine*, *triglyceride*, and *cholesterol* (HDL ratio). The metabolomics biomarker domain (*n* = 2) consisted of two subpanels—*up-regulated* (higher levels representing increased risk) and *down-regulated* (lower levels representing increased risk)—from the metabolite panel that distinguished PDID from PDND (Han et al., 2017) see further description of metabolite biomarker protocols in 2.5. The neurocognitive domain (*n* = 5) included indicators spanning memory (*CVLT immediate word recall* (Elwood, 1995), executive function (*Trail A* and *Trail B* (Reitan, 1986), and processing speed (s*imple reaction time* and *four choice reaction time* (de Frias et al., 2012); see (Dixon et al., 2007) for speed data correction procedures). The psychological and lifestyle assessments domain (*n* = 7) included *depressive symptoms* measured by the Geriatric Depression Scale (Weintraub et al., 2006), *Neuropsychiatric inventory questionnaire* (*NPI*)*-sleep*, *NPI*-*anxiety* (Cummings et al., 1994), *activities of daily living* measured by the Schwab and England Activities of Daily Living-ON (self-reported activities when medications are working (Schwab, 1969), *body mass index* (*BMI*), *SMMSE* (Molloy and Standish, 1997), and the *MoPaRDS* (Dawson et al., 2018; Bohn et al., 2023).

### 2.4. MRI protocol

Magnetic resonance imaging (MRI) scans were acquired using a Siemens Sonata 1.5T scanner and automatically processed using the *FreeSurfer* 6.0. Automated segmentation of subcortical volumes and ventricles was performed using the *FreeSurfer* image analysis suite (Fischl et al., 2002) freely available for download (FreeSurfer, 2017). *FreeSurfer* was run on the Canadian Brain Imaging Research Platform (CBRAIN), which is web-based software for distributed computing intended for neuroimaging research (Sherif et al., 2014). A full description of imaging procedures are documented elsewhere (Camicioli et al., 2011; Dadar et al., 2020). Estimated intracranial volume from the aseg file were used (Fischl et al., 2002). Cortical thickness was the average thickness of all measured brain areas (*n* = 68).

### 2.5. Metabolomics biomarker protocol

Blood samples were collected from all participants at baseline. Pairwise metabolomics analyses were previously conducted with the chemical isotope labeling liquid chromatography mass spectrometry (CIL LC-MS) technique using a Bruker maXis impact high-resolution quadrupole time-of-flight mass spectrometer with electrospray ionization (Bruker, Billerica, MA) combined with an Agilent 1,100 HPLC (for further details see (Han et al., 2017). Two metabolite panels were developed from the original work, such that one up-regulated (i.e., higher metabolite levels) and the other down-regulated (i.e., lower metabolite levels) indicate increased risk of belonging to the PDID group. The up-regulated panel consisted of five metabolites observed in higher concentrations in the PDID group (i.e., Hydroxy-isoleucine, His-Asn-Asp-Ser, Alanyl-alanine, Putrescine [-2H], 3,4-Dihydroxyphenylacetone). The down-regulated panel consisted of three metabolites observed in lower concentrations in the PDID group (i.e., Riboside of Purine [ + O], Desaminotyrosine, Purine [ + O]). All the metabolite measurements in this dataset were relative concentrations with respect to a reference sample, which represented the averaged metabolite concentration level across all samples (control + PDND + PDID). We then conducted sample-wise normalization to correct systematic inter-sample differences. Although no metabolite-wise scaling was performed, this is not a requirement for variables used in RF models. Each subpanel consisted of the additive score of the averaged experimental duplicates for each normalized metabolite values for each participant.

### 2.6. Analytic approach

The current combinatorial signature was undertaken as a manual preprocessing step. Specifically, (a) for feature selection we included variables based on their relevance to cognitive decline or dementia and removed irrelevant or redundant variables, and (b) for data reduction we used variable aggregation (e.g., mean of gait measures).

**FIGURE 1:**
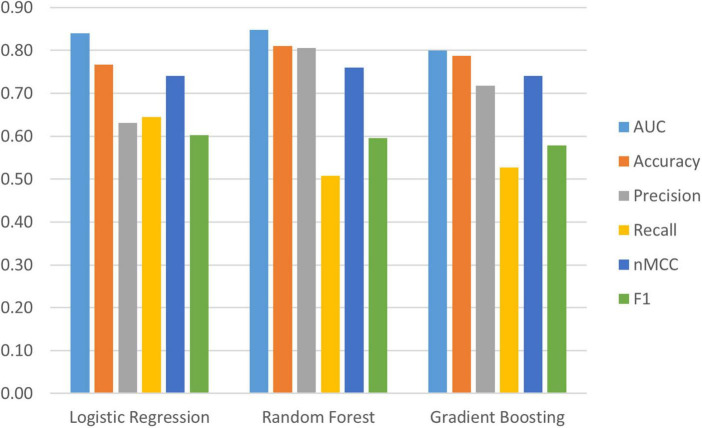
Evaluation metrics for three machine learning models; Logistic Regression, Random Forest Classifier, and Gradient Boosting. Random Forest has the best evaluation metrics and has been retained as the best model for the current research; Area Under the Curve (*AUC*) = 0.85 (95% confidence interval (CI) [0.83, 0.86]), Accuracy = 0.81 (95% CI [0.80, 0.82]), Precision = 0.81 (95% CI [0.75, 0.86]), Recall = 0.51 (95% CI [0.46, 0.55]), normalized Matthews Correlation Coefficient (n*MCC*) = 0.76 (95% CI [0.74, 0.78]), F_1_ = 0.60 (95% CI [0.56, 0.63]).

**FIGURE 2:**
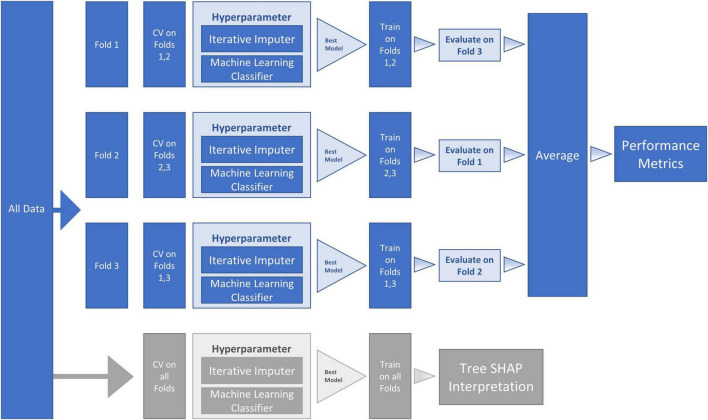
Machine Learning (ML) Pipeline for Random Forest (RF) classifier model and SHapley Additive exPlanation (SHAP) model. The workflow proceeds within the ML pipeline with internal (columns four and five) and external (columns six and seven) cross-validation (CV). As represented in the second and third columns, the dataset was sequentially divided into three folds—with each fold being used for testing one time, thus producing three CV analyses. Two sequential steps were conducted at each of the three fold splits (a) missing data imputation and (b) hyperparameter tuning. The hyperparameter boxes represent tuning that was conducted by performing internal CV on the training folds to find the best model (the model with the highest Area Under the Curve [*AUC*]). The best model (with selected hyperparameters) was then fitted on the training folds and evaluated on the testing fold. The average of the three fold splits (column eight) was used to estimate the performance metrics of the final tuned model fitted on all the data. To reduce variance due to the small sample size, this procedure was repeated 10 times and averaged to obtain final performance metrics (column nine). The lower row in the figure (in gray) represents the SHAP steps used for model interpretation. Specifically, we used *TreeExplainer* to approximate the original model and calculate Tree SHAP values that were used for the interpretation plots.

We used Python (2020) (3.7.6) and compared three machine learning algorithms from the s*cikit-learn* package (Pedregosa et al., 2011): (a) *LogisticRegression*, (b) *RandomForestClassifier* and (c) *GradientBoostingClassifier* to identify important (out of 38) baseline risk and biomarker predictors of PDID (vs. PDND). Comparing the model metrics (see Figure 1) and the substantially shared leading predictors of these models we identified RF as the best model for the current data. RF is an ensemble method (see Figure 2 for work flow) that combines multiple decision trees through majority voting and is an optimal technique used to simultaneously test a large number of predictors (Tseng et al., 2020). As a recursive partitioning multivariate data exploration technique, RF combines predictions across multiple classification and regression trees, each based on a random sample of participants and predictor variables. To accommodate a small and unbalanced sample, we used stratified *k*-fold cross-validation to evaluate the RF model for both internal and external validation (Hastie et al., 2009; Pedregosa et al., 2011). Because smaller sample sizes preclude a testing-training split (i.e., subsets would be too small), the *k*-fold technique provides the mechanism for examining a series of training and testing subsets within the model. Therefore, we used a *3*-fold cross-validation procedure that sequentially divided the dataset into three folds (or subsets). The sequence of analyses includes two folds used for training (internal validation) and one fold used for testing (external validation); this repeats until each fold has been used once for testing. To account for the unbalanced sample, stratified *k*-fold cross-validation ensures that each fold contains approximately the same proportion from each class as the overall sample. Specifically, each fold contained 71% from PDND and 29% from PDID. The best hyperparameters for the RF model were selected by internally evaluating each combination of hyperparameter values from the provided options (e.g., num_trees 100, 500, or 1,000) using the k-fold technique on the training folds. The best model for each fold split was then fitted on the training folds and evaluated on the testing fold. This was done for each fold split and the average performance was used to estimate the performance of the final tuned model fitted on all the data. This procedure was repeated 10 times and averaged to reduce variance due to the small sample size.

#### 2.6.1. Missing data

The baseline data included 2.3% overall missing predictor values, with specific variable missingness ranging from 2 to 12.5% (see Table 1 for percentage of missing data). For missing data estimation, we used *IterativeImputer* (s*cikit-learn* package (Pedregosa et al., 2011)). Specifically, *BayesianRidge*, the default estimator used for *IterativeImputer*, imputes missing values for any predictor as a function of all other predictors by using regularized linear regression. Imputation begins with the predictor with the least missing data and progresses to the predictor with the most missing data.

#### 2.6.2. RF Classifier Model

We used the *sklearn pipeline* (s*cikit-learn* package (Pedregosa et al., 2011)) that is designed to assemble several steps that can be cross-validated together. In this study, two sequential steps were conducted at each fold: (a) missing data imputation and (b) RF classifier (final parameters: n_estimators = 100, criterion = gini, max_depth = None, max_features = sqrt). The advantage of this approach is that missing data are imputed within each cross-validation fold thus avoiding data leakage issues (i.e., training the model with data from outside the training data set, such as the testing dataset).

#### 2.6.3. Reporting results of data-driven analyses

We report the results of the RF classifier model in two phases of data-driven analyses: (a) general analytic model evaluation of model fit and (b) follow-up determination of the most important predictors contributing to the observed classification.

##### 2.6.3.1. Analytic model evaluation metrics

The analytic model evaluation metrics are averaged across the 10 trials of the external 3-fold cross-validation. The principal metric for evaluating the general classification (RF) is the (*AUC*). *AUC* represents the ability of the model to distinguish between two (unobserved) clinical classes at baseline (i.e., PDID, PDND). For interpretation, *AUC* values of 0.7 to 0.8 are considered acceptable, 0.8 to 0.9 are considered excellent, and above 0.9 are considered outstanding (Mandrekar, 2010). In this study, an AUC value in this range would constitute a model fit sufficient to move to the phase of evaluating the relative importance of the predictors. We also calculated five subsidiary indicators known to be sensitive to specific fit characteristics in the context of varying sample features (e.g., group sizes and balance). These values are reported in the results and interpreted for their specific indications in the discussion. *Classification accuracy* represents the percentage of baseline classifications that correctly predict the 3-year outcome diagnosis (i.e., PDND and PDID). It is calculated as the fraction of true positives and true negatives among all model classifications; value range = [0–1], higher values denote better classification. A *precision* metric is calculated as the fraction of true positives among all model classified positives [i.e., true positives/(true positives + false positives)]; value range = [0–1], higher values denote better precision. A *recall* metric is a sensitivity measure that is calculated as the fraction of model classified positives among the true number of positives [i.e., true positives/(true positives + false negatives)]; value range = [0–1], higher values are considered better. An *F*_1_ score is the harmonic mean of precision and recall. As such, it represents a useful additional evaluation metric in the case of small and unbalanced samples; value range = [0–1], higher values are considered more accurate for majority and minority classes. Normalized *Matthews Correlation Coefficient* (n*MCC*) is a metric that considers all categories of the confusion matrix and is considered a reliable metric for evaluating binary classification, especially for imbalanced datasets; value range = [0–1], higher values are considered to make correct predictions for both the majority and minority classes (Chicco and Jurman, 2020).

##### 2.6.3.2. Integrating Tree SHAP values for interpretation of important predictors in the RF classifier model

###### 2.6.3.2.1. Tree SHAP values

We estimated Tree SHAP values using *TreeExplainer* (Lundberg et al., 2018, 2020). Tree SHAP is an adaptation of classic Shapley values that enhances interpretability of complex tree-based machine learning prediction models (e.g., RF) using a simpler model as an interpretable approximation of the more complex model (Shapley, 2001; Molnar, 2019; Lubo-Robles et al., 2020; Rodríguez-Pérez and Bajorath, 2020). Specifically, Tree SHAP values (a) are computed using conditional expectations combined with the original Shapley values to attribute refined values for each predictor, (b) take both main (one predictor) and interaction (all coalitions of predictors) effects into account, (c) are based on the magnitude of predictor attributes rather than decreases in model performance associated with permutation importance, and (d) provide unique additive feature importance that adhere to local accuracy, missingness, and consistency (Lundberg et al., 2018). Local interpretable model-agnostic explanations (LIME) was not used as an explanation model due to the (a) linear model approach that can lead to instability of explanations and (b) assumptions of variable independence (Elshawi et al., 2019; Gaur et al., 2022a,b). A common assumption in aging neuroscience is that there are expected interactions among PD-related predictors of future dementia status (Dixon and Lachman, 2019).

###### 2.6.3.2.2. Displaying results as Tree SHAP Plots: representation and interpretation

In the results section, we include three Tree SHAP plots that provide complementary visual representations of the obtained prediction model (Lundberg et al., 2018, 2020; Molnar, 2019). The first figure presents a global feature importance plot that shows the average absolute Tree SHAP values for each predictor in descending order of importance. The remainder of the figure includes the composition (horizontal bars) and cumulative (curved line) ratios that depict each predictor’s contribution to the total model. The second figure presents a Tree SHAP summary plot. This plot displays the combination of predictor importance with predictor magnitude, prevalence, and direction of effect. Such summary plots show the relationship between the value of the predictor and the specific impact it has on the prediction (i.e., increases or decreases the prediction). Each participant’s Tree SHAP value for each predictor is represented by a point on the plot. The position on the y axis is determined by the predictor and on the x axis by the Tree SHAP value; positive values indicate membership in the PDID group, negative values indicate membership in the PDND group. The color of the points represents the value of the predictor from low (blue) to high (red) showing the distribution of the Tree SHAP values per predictor and the direction of the effect. The third figure includes 10 Tree SHAP dependence plots. They are designed to show the exact form of the predictor relationship. Specifically, the predictor value on the x axis influences the corresponding Tree SHAP value on the y axis; the point on the x axis where the corresponding Tree SHAP value exceed 0 indicates the threshold value related to risk.

## 3. Results

Two phases of data-driven analyses were conducted: (a) testing the overall RF classifier model fit and (b) utilizing Tree SHAP for deeper interpretation of the most important predictors contributing to the observed classification. In the first phase, we simultaneously tested 38 multi-modal predictors to discriminate within a baseline PD cohort two subtypes defined by later diagnoses as being PDND or PDID. The *AUC* is the main metric for evaluating the overall classification model fit. According to the stipulated standards, the observed fit can be characterized as excellent (*AUC* = 0.85 (95% confidence interval (CI) [0.83, 0.86]). We note that several subsidiary indicators, known to be sensitive to specific fit characteristics in the context of varying sample features (e.g., group sizes and balance), provide complementary information. The observed metrics are: *classification accuracy* = 0.81 (95% CI [0.80, 0.82]); *precision* = 0.81 (95% CI [0.75, 0.86]); *recall* = 0.51 (95% CI [0.46, 0.55]); *F*_1_ score = 0.60 (95% CI [0.56, 0.63]); and n*MCC* = 0.76 (95% CI [0.74, 0.78]). Taken together, the *AUC* and supplemental indicators demonstrate a prediction model that efficiently distinguishes between unobserved classes—PDID from PDND at a time point 3 years prior to the dementia diagnosis.

In the second phase, we examine the full complement of predictors in order to determine (a) their relative importance (leading or trailing) in producing the excellent classification model fit, (b) the specific direction of influence for predictors, and (c) the magnitude, or risk threshold, of the leading predictors. Three Tree SHAP plots display specific predictor-related model performance.

**FIGURE 3:**
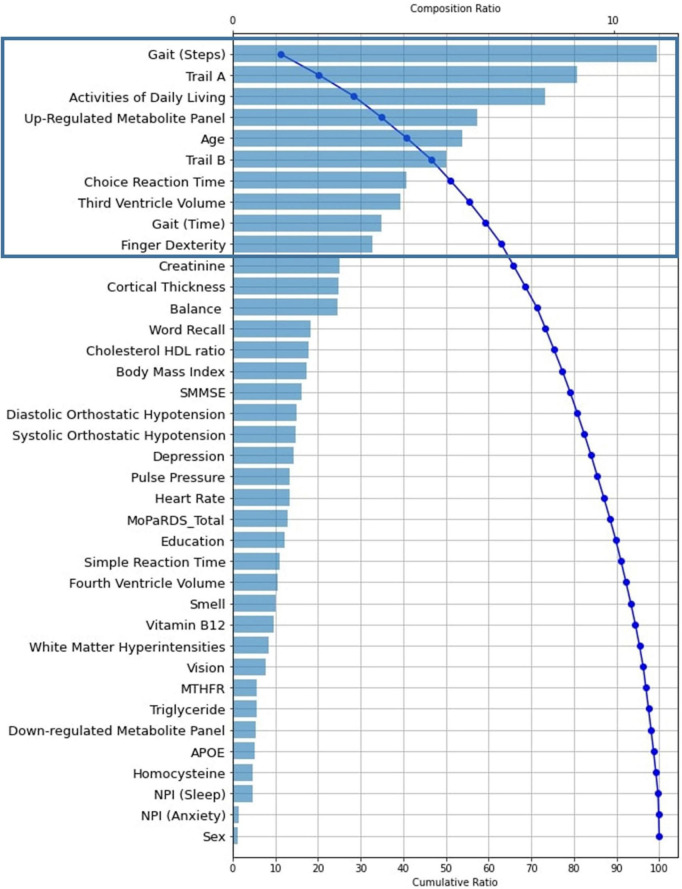
Tree SHAP Plot of Global Feature Importance indicating the composition and cumulative ratios for the 38 baseline predictors tested in the RF classifier model (*AUC* = 0.85, Accuracy = 0.81, Precision = 0.81, Recall = 0.51, n*MCC* = 0.76, F_1_ = 0.60). Predictors are plotted as their individual composition ratio (blue bars; scale shown at the top of the plot) in descending order of importance. Composition ratio is the amount the predictor contributes to the model output. The blue curved line reflects the cumulative ratio with each added predictor (scale shown at the bottom of the plot). The line arcs from approximately 12% for the most important predictor [gait (steps)] to 100% for the least important predictor (*APOE*). For example, creatinine (the final predictor indicated in the black rectangle) has a composition ratio of approximately 4 (explains 4% of the model; as indicated by the blue bar and scaled by the top x axis) and also identifies the cumulative ratio of the 10 most important predictors as 62.5 (i.e., together they explain 62.5% of the model, as indicated by the blue curved line and scaled by the bottom x axis). RF, Random Forest; *AUC*, Area Under the Curve; n*MCC*, normalized Matthews Correlation Coefficient; *APOE*, Apolipoprotein E.; SMMSE, Standardized Mini Mental State Exam; MoPaRDS, Montreal Parkinson Risk of Dementia Scale; NPI, Neuropsychiatric Inventory Questionnaire.

In Figure 3, the global feature importance plot shows the predictors listed in descending order of importance. Blue bars indicate the composition ratio of each predictor. The curved blue line shows the cumulative ratio of each predictor and its predecessors. For example, the most important predictor gait (steps) shows a 11% composition ratio. The cumulative ratio line starts there at 11% and progresses to 100% for the least important predictor (sex). We focus on the 10 leading predictors of this model because (a) there is an evident elbow (or break in the distribution) at this point, (b) these predictors explain a substantial amount of the model, and (c) all predictor contributions to the model after this point are ≤ 3%. The 10 leading predictors explain 62.5% of the model, whereas the remaining 28 predictors account for 37.5% of the model. Specifically, the 10 leading baseline predictors of later PDD listed in descending order of importance (% of model explained) are: gait (steps, 11%), Trail A (8.9%), activities of daily living (8.1%), up-regulated metabolite panel (6.4%), age (6.0%), Trail B (5.6%), choice reaction time (4.5%), third ventricle volume (4.4%), gait (time, 3.9%), and finger dexterity (3.6%).

**FIGURE 4:**
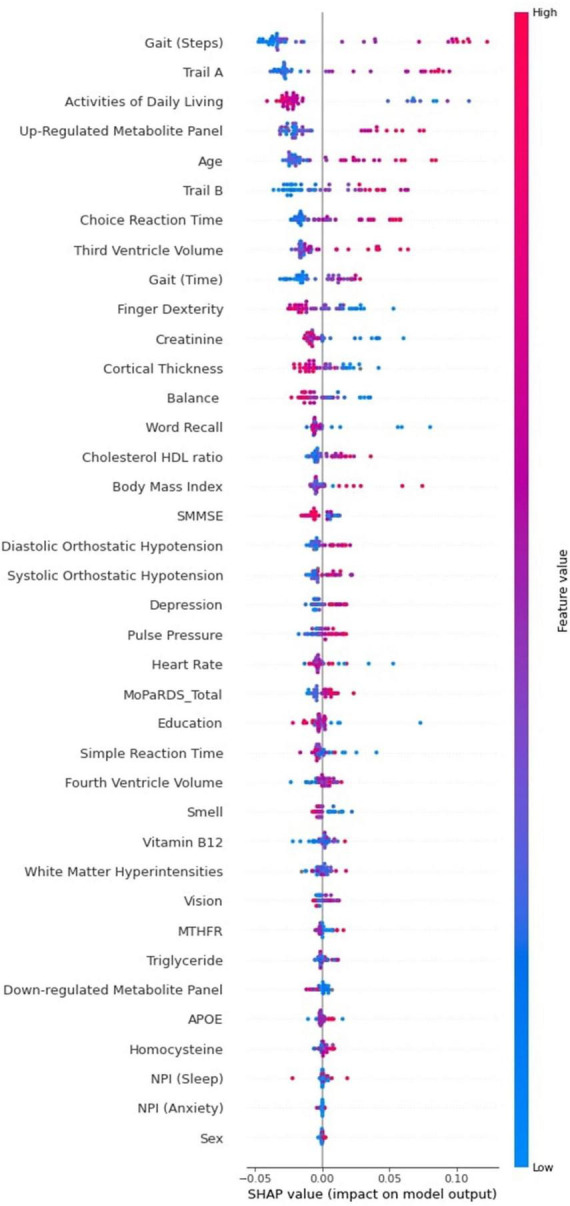
Tree SHAP Summary Plot for the 38 baseline predictors of PDID (vs. PDND) tested in the RF classifier model. The interpretation of this plot is based on three factors. First, in the distribution of the points on the summary plot, each dot represents one participant’s Tree SHAP for each predictor. Second, the position of the dots on the x axis relates to the prediction effect. In this case, belonging to the PDID group is indicated by a positive Tree SHAP and the more positive the Tree SHAP the greater the dementia risk for baseline PD patients. Third, the color of the dots indicates the direction of the effect for each predictor (red indicates higher values; blue indicates lower values). For example, the leading PDID risk predictor gait (steps) has a long tail to the right made up of red dots indicating that worse gait (greater number of steps) at baseline increases the risk of belonging to the PDD group three years later. It is also important to note that some predictors lower in importance also have long tails to the right. For example, high BMI does not affect later dementia for many PD patients at baseline, but it increases risk of dementia for some individuals. Additional personalized approaches can determine which of these persons with high BMI are at elevated risk for later dementia. PDID, Parkinson’s Disease Incipient Dementia; PDND, Parkinson’s Disease No Dementia; RF, Random Forest; PD, Parkinson’s Disease; PDD, Parkinson’s Disease Dementia; BMI, Body Mass Index.

The Tree SHAP summary plot is presented in Figure 4. This plot provides detailed information pertaining to predictor magnitude, prevalence, and direction of effect. We again focus on the 10 leading predictors of PDID. Specifically, the figure indicates that these leading baseline predictors of later PDD can be further characterized in terms of direction of effects. In order, the display shows the following effect directions: (a) worse gait (greater number of steps), (b) more time to complete the Trail A task, (c) fewer activities of daily living, (d) higher level of the up-regulated metabolite panel, (e) older age, (f) more time to complete the Trail B task, (g) more time to complete the choice reaction time task, (h) larger third ventricle volume, (i) slower gait, and (j) poorer finger dexterity (lower number of finger taps).

**FIGURE 5:**
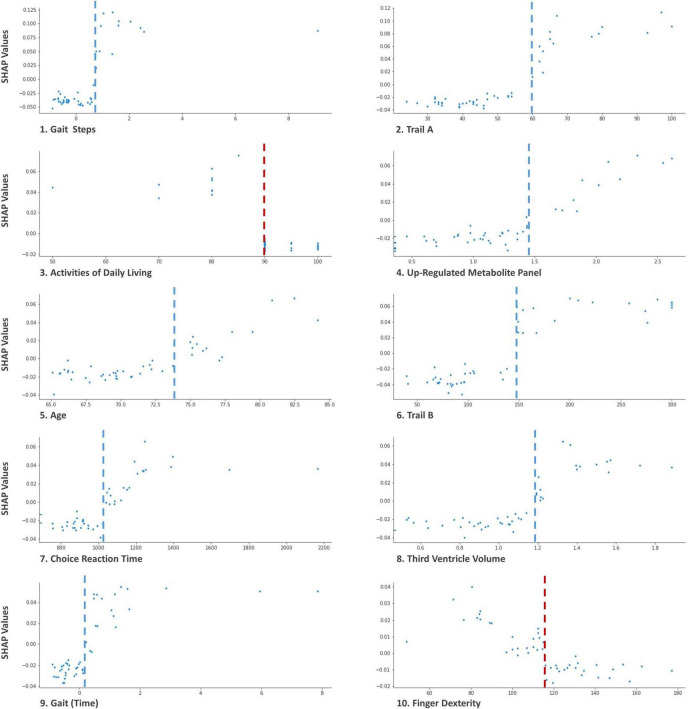
Tree SHAP Dependence plots for the 10 leading baseline predictors of PDID. These plots display the threshold at which the risk for each predictor is exacerbated. Predictor values are represented on the × axis and Tree SHAP values attributed to the predictor are represented on the y axis. Each point represents one participant. For gait (steps; 1), Trail A (2), up-regulated metabolite panel (4), age (5), Trail B (6), choice reaction time (7), third ventricle volume (8), and gait (time; 9) values on the right side of the gray dashed line indicate increased risk of PDD. For activities of daily living (3) and finger dexterity (10) values on the left side of red dashed line indicate increased risk of PDD. For example in panel 1, as the number of steps for gait at baseline increases (x axis), Tree SHAP values increase (y axis). The threshold of PDD risk elevation for this leading predictor is identified as the point at which the number of standardized steps at baseline approaches 1.0, Tree SHAP values > 0 (visually indicated by dashed blue line). The text identifies the actual threshold values for each of the predictors.

Figure 5 shows Tree SHAP dependence plots for the 10 most important predictors. Not only do Tree SHAP dependence plots allow us to see the direction of risk but also the threshold at which risk is increased. For each baseline predictor, Tree SHAP values above 0 indicate increased risk of PDD within 3 years. Panel 1 of Figure 5 shows the results for gait (steps). As the number of standardized steps reaches a threshold of ≥ 1 (shown on the x axis), Tree SHAP values (shown on the y axis) become increasingly positive, indicating substantial prediction of PD patients later diagnosed with PDD. The threshold value for each of the 10 leading predictors in the plots included in this figure are as follows: (a) gait ≥ 0.8 standardized steps, (b) Trail A performance exceeding 60 ms to complete the task, (c) activities of daily living ≤ 90% independence, (d) level of the up-regulated metabolite panel ≥ 1.4, (e) age ≥ 74 years old, (f) Trail B performance ≥ 150 ms to complete the task, (g) choice reaction time performance ≥ 1,050 ms to complete the task, (h) third ventricle volume ≥ 1.2 cm^3^, (i) gait measured as standardized time to complete task of ≥ 0.15 s, and (j) < 150 finger taps. In the figure, these threshold values are represented by gray (increased risk to the right of the line) and red (increased risk to the left of the line) dashed line in the distributions of every panel.

Cross-model validation analyses comparing RF and GB showed that of the leading 10 predictors described above for RF, eight were also in the leading 10 for GB. These common eight predictors of (later) PDD were: gait (steps), Trail A, activities of daily living, up-regulated metabolite panel, age, Trail B, choice reaction time, and third ventricle volume. In addition, the ninth and tenth RF predictors ranked in the top 15 of the GB model and the ninth and tenth GB predictors ranked in the top 14 of the RF model. We note the additional predictors (i.e., word recall, creatinine) as contributed by the GB model.

## 4. Discussion

Not all diagnosed PD patients develop dementia and those who convert do so at different intervals and may do so as a function of differentially accumulating risk across multiple domains (Guo et al., 2021). With a well-characterized clinical sample of recently diagnosed PD patients, we demonstrate the usefulness of Python-based machine learning prediction models (RF) to discriminate, at baseline, those dementia-free PD patients who were diagnosed with dementia 3 years later (PDID) from those who remained dementia free (PDND). The prediction model included 38 multi-modal dementia risk factors. The analyses produced excellent classification results (*AUC* = 0.85). We identified 10 predictors of leading feature importance in discriminating at baseline PDID from PDND, which collectively explained 62.5% of the RF classifier model. We used Tree SHAP applications to interpret the direction and magnitude of risk for these 10 leading predictors. Most complex machine learning classifier approaches have been described as *black-box* models because they predominantly focus on prediction accuracy as the main performance metric (Lundberg and Lee, 2017). The recent artificial intelligence-based development of the Tree SHAP application allows a deeper interpretion of the models in regard to the direction of predictor effects and magnitude of risk (see Figures 3, 4). The 10 identified leading predictors have been empirically associated with dementia outcomes for PD patients, and many of them have been reported by investigators in other prediction models (Liu et al., 2017; Schrag et al., 2017; Dawson et al., 2018; Marinus et al., 2018; Phongpreecha et al., 2020; Guo et al., 2021). Collectively, these variables spanned 6 of the 10 risk domains considered in the present study. Below we discuss the 10 leading predictors of subsequent PDD that were identified in the competitive context of machine learning, organized by their respective domains, and interpreted by the Tree SHAP approach.

### 4.1. Gait and motor function domain

Important predictors from the gait and motor function domain included two gait measures and one motor function measure: (a) number of steps, ranked as the most important predictor, (b) time to complete a walking task, ranked as the ninth, and (c) finger dexterity, ranked as the tenth most important predictor. First, more steps and longer time to complete the walking tasks at baseline predicted an increased risk of PDD at follow-up (i.e., belonging to the unobserved PDID group). The gait risk characteristics may be an early indication of the shuffling walk that is a clinical predictor of PD and the resulting postural-instability-gait disorder. Indeed, a recent meta-analysis reported that postural-instability-gait disorder predicted PDD (Guo et al., 2021). Previous associations between mobility and cognitive decline and MCI in PD have been reported (Brandão et al., 2020). In contrast, balance and gait velocity have demonstrated associations with risk of dementia in persons without PD but not in patients with PD (Horne et al., 2021). Nevertheless, our study has identified these variables as leading predictors, one of which is the most important, from a 38-item risk factor inventory. Specifically, we found that the gait measures accounted for 15% of the model with number of steps being the most important predictor that accounted for 11%. Our findings show that these basic measures of gait contributed to the prediction of membership in the PDID group, suggesting that more complex measures of gait disorders may not distinguish PDND from PDID until closer to dementia diagnosis. These findings add evidence to the literature that reported posture and gait measures, but not bradykinesia and tremor measures, predicted dementia (Domellöf et al., 2015). Second, a slower rate of finger tapping (fewer finger taps per minute) predicted membership in the unobserved PDID group at baseline. Although little research has been reported for finger dexterity in regard to PDD, hand dexterity, measured by the nine-hole peg test, has previously been reported to be slower for Lewy body dementia and PDD than for those with PD (Fritz et al., 2016).

### 4.2. Neurocognitive domain

Leading predictors from the neurocognitive domain included Trail A (ranked second), Trail B (ranked sixth), and choice reaction time (ranked seventh). Specifically, increased time to complete these three neurocognitive speed measures predicted membership in the PDID group. These neurocognitive speed measures have previously been identified as predictors of PDD. Specifically, in a group of PD patients with MCI at baseline, attention and mental flexibility (i.e., Trail A, Trail B) as well as measures of episodic memory, visuospatial function, and verbal fluency predicted conversion to PDD (59%) within 5 years (Domellöf et al., 2015). A study identifying global cognition, verbal fluency, memory, and attention as predictors of PDD, reported that choice reaction time declined faster in a PDID group over 5 years compared to a PDND group (Lawson et al., 2021). When MCI was controlled for at baseline, choice reaction time became a predictor of developing PDD at follow-up. A risk scale including neurocognitive measures (*AUC* = 0.90) out-performed an alternate risk scale using only biological measures (*AUC* = 0.71) in distinguishing PDID from PDND (Phongpreecha et al., 2020). Further investigation of early signals of exacerbated cognitive decline in non-demented PD patients may contribute to differentiating those with elevated PDD risk.

### 4.3. Metabolomics biomarker domain

The remaining four domains represented in the 10 leading predictors consisted of one predictor each. In the metabolite domain, the up-regulated metabolite panel, consisting of five metabolites, ranked as the fourth most important predictor. Higher levels of our baseline up-regulated metabolite panel predicted PDID. Our panel was based on an earlier comprehensive metabolomics analyses of an 8-metabolite panel that discriminated PDID from PDND patients at baseline with 86% accuracy (Han et al., 2017). The five metabolites in our up-regulated metabolite panel included Hydroxy-isoleucine, His-Asn-Asp-Ser, Alanyl-alanine, Putrescine [-2H], 3,4-Dihydroxyphenylacetone. Hydroxy-isoleucine, an oxidized end product of leucine or hydroperoxyleucines, may be a useful marker of protein oxidation leading to cellular protein and membrane damage (Yu et al., 2013). Protein oxidation has been associated with aging and a number of aging related diseases.

### 4.4. Demographic domain

An important predictor of PDID in the demographic domain was age (ranked fifth). Not surprisingly, older chronological age predicted PDID membership. Previous research has implicated older chronological age to be among the most consistent predictor of PDD (Horne et al., 2021). Younger age of onset of PD symptoms has also been identified as a predictor of PDD (Brandão et al., 2020; Oxtoby et al., 2021). Our findings show that chronological age remained a leading predictor even when tested in large inventory of risk factors.

### 4.5 Psychological and lifestyle assessment domain

In the broader research area of dementia risk in aging, growing research attention has been directed at determining the relevance of engagement in cognitive, physical, and social activities in delaying or preventing dementia (Anstey et al., 2019; Dixon and Lachman, 2019; Livingston et al., 2020). The results are promising but mixed and most often reported in the context of Alzheimer’s disease dementia. In the present study, one marker representing this general domain was the Schwab and England Activities of Daily Living Scale that measures independence in performing daily activity chores. This feature was the third most important predictor. We observed a prediction direction that is consistent with the approach featured in the broader dementia risk literature, viz., lower levels of activities were associated with membership in the unobserved baseline PDID group.

### 4.6. Neuroimaging domain

Larger third ventricle volume at baseline ranked as the eighth leading predictor of increased likelihood of belonging to the PDID group (and converting to dementia after 3 years). There has been debate about the usefulness of ventricle volume in particular and imaging markers in general for prediction of PDD. Although some previous research has implicated ventrical measures in the development of PDD (Camicioli et al., 2011; Dong et al., 2017), the evidence to date has not be strong enough for ventricle volume to be considered a biomarker but rather as an assessment tool to be used in the presence of other markers (Behnke et al., 2019). Our results contribute evidence in favor of using third ventricle volume as a biomarker. A recent review of the ability of neuroimaging to predict dementia in PD concluded that imaging markers were not currently sufficient to accurately predict dementia, although this may change with the development of more sensitive imaging techniques in combination with other dementia risk markers (Lanskey et al., 2018). Abnormal accumulation of brain proteins associated with PD (e.g., Lewy body accumulation or AD-related pathology such as amlyloid β and tau) may account for more widespread cortical and subcortical atrophy and therefore ventricular enlargement (Dong et al., 2017).

### 4.7. Non-predicting dementia risk domains

We note four dementia risk domains that were not represented by markers in the leading predictor group: cardiovascular, candidate biomarker domain, genetic, and sensory. Risk factors associated with these four domains were (a) previously associated in one or more studies with PDD outcomes and (b) represented in the current computationally competitive analyses as among the trailing predictors. We recommend that representative markers from these domains continue to be used in clinical screening work or in larger-sample validation studies. Among the additional and occasionally observed dementia risk predictors most noticeably missing from the leading cluster in this study were: (a) orthostatic hypotension (Anang et al., 2014; Guo et al., 2021), (b) REM sleep disorder (Dawson et al., 2018), (c) sex (Dawson et al., 2018; Phongpreecha et al., 2020), and (d) *APOE* (Cereda et al., 2016).

### 4.8. Strengths and limitations

There are several strengths and limitations associated with our study. First, this was a longitudinal study with a well characterized, homogenous sample of initially non-demented PD patients (Camicioli et al., 2011; de Frias et al., 2012; Hussain and Camicioli, 2018; McDermott et al., 2018; Sapkota et al., 2018). We assembled a large number of potential baseline risk factors and biomarkers (>35) of PDD with relatively complete data (2% overall missing data). Second, we used a powerful data-driven protocol that integrated machine learning analytics (i.e., RF classifier) with artificial intelligence interpretion models. This approach is well suited to larger and smaller sample sizes and is designed to accommodate multiple predictors and high-dimensional data. Specifically, the RF classifier is a recursive partitioning multivariate data exploration technique that averages and incorporates multiple diverse classification models, effectively reduces overfitting of the training data, and results in good accuracy for the testing data (Strobl et al., 2009; Yang et al., 2010). Third, RF classifier models provide limited interpretation of specific predictors (Lundberg et al., 2020). Accordingly, we used Tree SHAP—a computationally efficient approach to providing deeper interpretations than typically available in RF classification models (Lundberg and Lee, 2017). By evaluating a unique solution, the Tree SHAP approach allowed us to interpret each of the leading predictors in terms of their relative importance, direction of risk, and magnitude of risk threshold. Although there are other model explanation approaches that could have been used, Tree SHAP provides a strong and complete summary of the model characteristics (Covert et al., 2021; Mitchell et al., 2022). Specifically, Tree SHAP builds on previous methods to produce a unified framework that compared favorably with alternatives such as DeepLIFT or LIME and was the only additive feature attribution method that did not violate local accuracy or consistency (Lundberg and Lee, 2017; Chen et al., 2021).

Fourth, as noted, a limitation is that these analyses were conducted on a relatively small and unbalanced sample. We compared Linear Regression, GB, and RF algorithms. RF provided the best model metrics. The analytic approaches we used have been established as suitable for a wide range of samples varying in characteristics and number of predictors. In the case of the RF algorithm, this accommodation is accomplished by its low vulnerability to noise and overtraining (Breimen, 2001; Thanh Noi and Kappas, 2017). Furthermore, the model evaluation was conducted with stratified 3-fold cross-validation which is recommended for smaller and unbalanced samples over the standard training, validation, and testing splits (Hastie et al., 2009). Specifically, the 3-fold cross-validation method maintained group proportions to accommodate the unbalanced sample (i.e., 71% from PDND and 29% from PDID). Model metrics were estimated from testing each fold. Fifth, an issue that occurs with unbalanced samples is that commonly used model evaluation metrics may be over-estimated (Brownlee, 2020). In the present unbalanced study, there may be a bias toward classification in the majority group (PDND) resulting in an overestimation of accuracy. As reported, we examined the recall value, an indicator of membership in the minority group (PDID) that is more likely to be low in unbalanced samples. However, normalized *Matthews Correlation Coefficient*, a reliable metric for small and unbalanced samples (Chicco and Jurman, 2020), was strong. Although all other model indicators were strong, the observed moderate recall value underscores a recommendation for further replication in larger or more balanced samples.

## 5. Conclusion

Dementia is a clinically impactful non-motor outcome of PD. There is considerable heterogeneity in how rapidly and broadly dementia develops after a diagnosis of PD. Early identification of those most at risk—as well as characterization of the leading dementia predictors—is an important priority of PD research. The present well-characterized clinical sample of newly diagnosed and non-demented PD patients allowed us to test a large battery of 38 dementia risk factors in a single RF classifier model. From this full collection of candidate dementia predictors we identified 10 leading biomarkers and risk factors for PDD at 3-year follow-up. Although the present PD sample is small, our use of (a) powerful data-driven classification analytics combined with (b) cutting-edge Tree SHAP graphical interpretation provided valuable insights into the important risk characteristics that distinguished the two unobserved baseline PD groups (PDND, PDID). This study provides promising insights into potential mechanisms associated with emergence of dementia in persons with PD. The present omics-related results were promising. Future research should consider integration of other omics data (e.g., lipidomics, proteomics) as predictors of emerging dementia in PD. With replication and extension, new clinical indicators of modifiable targets for early risk detection and intervention can be validated.

## Data availability statement

The data analyzed in this study is subject to the following licenses/restrictions: Data available with a reasonable request. Requests to access these datasets should be directed to RC, rcamicio@ualberta.ca.

## Ethics statement

The studies involving human participants were reviewed and approved by the University of Alberta Health Ethics Review Board. The patients/participants provided their written informed consent to participate in this study.

## Author contributions

GPM, LB, RC, and RAD contributed to the conception and design of the study. MG, WH, LL, and RC contributed to the data curation and preparation. GPM, WH, and RAD contributed to the methodology. GPM performed the statistical analysis in consultation with LB, SD, HF, and RAD. GPM, SD, RC, and RAD contributed to the funding acquisition. RC and RAD contributed supervision. GPM and RAD wrote the initial drafts of the manuscript. All authors contributed to manuscript revisions; all authors read and approved the submitted version.
